# Mitochondrial complex I and V gene polymorphisms in type II diabetes mellitus among high risk Mizo-Mongoloid population, Northeast India

**DOI:** 10.1186/s41021-016-0034-z

**Published:** 2016-03-01

**Authors:** Freda Lalrohlui, Sunaina Thapa, Souvik Ghatak, John Zohmingthanga, Nachimuthu Senthil Kumar

**Affiliations:** Department of Biotechnology, Mizoram University, Aizawl, 796004 Mizoram India; Department of Pathology, Civil Hospital, Aizawl, 796001 Mizoram India

**Keywords:** Type 2 diabetes mellitus, Demography, Polymorphism, Mitochondrial genes, Genetic variability

## Abstract

**Introduction:**

The study was carried out to identify the polymorphisms in mitochondrial genes (ATPase and ND1) in type 2 Diabetes Mellitus (T2DM) from Mizo population and to correlate the involvement of demographic factors.

**Findings:**

In the present study, 58 patients and 50 healthy volunteers were considered. The mutations observed were mostly base substitutions and were similar as reported for other populations. Three mutations are unreported and were found to be novel polymorphisms for diabetic disease. One heteroplasmic variation (MT3970 C > T) was found in 36.36 % of samples. Subjects with excessive smoked meat consumption and customary habit of smoking (ORs: 4.92; 95 % CI: 0.96–25.21) were found to be more prone to T2DM. Mitochondrial genes sequence analysis revealed the genetic variability between the healthy and diabetic samples.

**Conclusion:**

Mitochondrial ATPase and ND1 gene polymorphisms may be involved in triggering the risk for T2DM.

**Electronic supplementary material:**

The online version of this article (doi:10.1186/s41021-016-0034-z) contains supplementary material, which is available to authorized users.

## Introduction

Mitochondrial dysfunctions are involved in ageing and age-related diseases such as Diabetes [1]. Complex I and V is one of several enzyme complexes necessary for oxidative phosphorylation [2]. Patients with large mtDNA mutations like deletion, deletion-duplication or in association with mtDNA point mutations generally in tRNA genes (tRNA (LEU(UUR)) has been reported with Diabetes Mellitus [3]. Na+,K + −ATPase is an ubiquitous membrane enzyme that allows the extrusion of three sodium ions from the cell and two potassium ions from the extracellular fluid. Abnormal accumulation of ROS activates UCP2, which in turn results in proton leak across the mitochondrial inner membrane leading to reduced b-cell ATP synthesis and content. This is a critical parameter in regulating glucosestimulated insulin secretion and release which ultimately increases circulating blood glucose level [4]. ND1 gene provides directives for making a protein called NADH dehydrogenase I. The actions of mitochondrial content are often reduced in patients with T2DM, or insulin resistance [5, 6]. Type II diabetes (T2D) is considered as the heterogeneous disease with altered insulin production by the pancreatic beta cell. The study of the relationship of ATPase and ND1 gene to type 2 diabetes has revealed the influence of the mitochondria on nuclear-encoded glucose transporters and the influence of nuclear encoded uncoupling proteins on the mitochondria [7]. There is evidence of a more global effect of mitochondrial dysfunction at the glucose transporter level and it will be interesting to study the variations in the genes involved among the different populations.

Earlier studies showed that mtDNA ND1 gene mutations at nt3310 (C > T), nt3667 (T > G) might contribute to the pathogenesis of DM with other genetic factors and environment factors [8, 9]. Howarth and Worsley [9] studied the dietary habits of elderly diabetics and have shown that faulty diet regimes can make the best of medicine ineffective.

Mizoram is one of the northeastern states of India, bordered by Bangladesh in the west and Myanmar on the east and south. Mizo people belong to the Mongoloid race and are ethnically and culturally most diverse tribe in the world [10]. Mizoram lies between 21°58′ & 24°35′ N latitude and 92°15′ & 93°29′E longitude and spread over 21,081 sq. kms area. Traditional Mizo food mostly comprises of boiled, stewed, smoked, steamed, or fermented form. Mizo food also comprises of certain leafy vegetables, fresh as well as preserved through smoking, such as mustard leaves (antam), pumpkin leaves (maian), beans leaves (behlawi), varieties of bamboo shoot (mautuai, rawtuai), fermented soya beans (bekang), fermented lard (sa-um) and dried fish chutney with green chilly. A peculiar habit of consumption of “tuibur” (tobacco smoke–infused aqueous solution) has been observed in Mizoram [11]. The present study was carried out to understand the influence of demographic factors on type 2 diabetics and associated mitochondrial polymorphisms in the Mizopopulation, North-east India.

## Materials and methods

### Sample collection and DNA extraction

A total of 58 patients with or without a family history of type 2 Diabetes Mellitus (median age 48 years, range 24–77) from Civil Hospital, Aizawl, Mizoram, India and 50 healthy volunteers (median age 48 years, range 35–63) were randomly recruited for this study from Mizo population. Senior diabetologist confirmed the diagnosis of Type 2 diabetes mellitus. The peripheral blood samples of these affected patients were kept in EDTA rinsed microcentrifuge tubes and stored in −20 °C freezer. Detailed information on demographic factors such as physical activity, dietary habits, previous disease history, alcohol and tobacco use and family history of diabetes were recorded during an in-person interview using a structured questionnaire (Additional file 1: Table S1). The ethical committee of all institutes approved the study protocol involved in the study. All volunteers were fully informed about the study and participated with their full consent. DNA extraction and quantification from the blood samples were performed according to Ghatak et al. [12].

### PCR amplification of ATPase and NDI gene

The mtDNA ATPase region (1046 kb) was amplified by PCR using primers KatPase-F (5′-CTAGAGCCCACTGTAAAGCTAAC-3′) and KatPase-R(5′-GAGCGTTATGGAGTGGAA GT-3′). Polymerase chain reaction (PCR) for ATPase region was carried out in 25 μl total reaction volume, each containing 100 ng of template DNA, 0.25 pM of each primer, 2.5 μl of 10X PCR buffer, 1.5 mM MgCl_2_, 200 mM dNTPs, and 1.5 U of Dream Taq green DNA polymerase (Fermentas, Germany). Polymerase chain reaction volume was heated initially to 95 °C for 5 mins. followed by 30 cycles each consisting of 1 min. denaturation at 95 °C, 40 s annealing at 56 °C, 1 min. extension at 72 °C. The reaction ended with a final extension step by incubating at 72 °C for 5 min. The entire mitochondrial ND1 gene spanning across nucleotide positions 3306 to 4261 was amplified. PCR amplification was performed using forward primer (5′-GAGCCCGGTAATCGCATAA-3′) and reverse primer (5′-GATAGGTGGCACGGAGAAT-3′). Polymerase chain reaction (PCR) for ND1 gene region was carried out in 25 μl total reaction volumes, each containing 100 ng of template DNA, 0.2 pM of each primer, 2.5 μl of 10X PCR buffer, 2 mM MgCl_2_, 200 mM dNTPs and 1 U of Dream Taq green DNA polymerase (Fermentas, Germany). The mixture was subjected to initial denaturation at 95 °C for 5 min., followed by 30 cycles of denaturation at 95 °C for 60 s, annealing of primers at 58 °C for 40 s, extension at 72 °C for 70 s. and a final extension cycle at 72 °C for 5 min. The PCR products were subjected to electrophoresis in a 1.2 % Agarose gel in 1X TBE buffer, stained with Ethidium Bromide, and images were obtained in GBOX gel documentation systems (UK) and sequenced.

### RFLP of PCR amplified product

The PCR amplified products were subjected to digestion using *AciI* (8–10 h at 37 °C), *Hae III* (3 h at 37 °C), *TaqI* (10–12 h at 56 °C) and *RsaI* (6–10 h at 37 °C in a total volume of 10 μl containing 3 μl of DNA, 0.4 μl of enzyme, 1 μl of buffer and 5.6 μl of water. The digested products were subjected to electrophoresis in 8 % PAGE (Polyacrylamide gel electrophoresis) gel at 40 V for 30 min and changed to 60 V, post staining was done using 1 μl of ethidium bromide and images were obtained in GBOX gel documentation systems.

### Sequence analysis

Based on the above digestion experiment, the polymorphic samples were selected and sequenced from both directions to ensure reading accuracy. Sequences and chromatograms obtained were examined using FINCH TV 1.4 software version (Geospiza. Inc., USA) and DNA Baser software version 4.16 and aligned by BLAST (http://www.ncbi.nlm.nih.gov/blast). All sequences were comparedwith the latest version of Revised Cambridge Reference Sequence (rCRS) and subsequently analyzed for the variation in sequences using Mito Tool Programming. The results of the DNA sequence analysis were compared with the published Cambridge Sequence using Mutation Surveyor version 1.4 DNA mutation analysis software (Softgenetics, State College, PA). Sequence differences between diabetic and healthy blood samples were recorded as mtDNA polymorphisms. Each polymorphism was verified against the Mitomap database (http://www.mitomap.org/) and further classified as novel or reported, depending on whether or not it is recorded in the database. The effect of amino acid substitutions based on the single nucleotide positions were predicted using Polyphen 2 software.

The number of base substitutions per site between sequences and averaging over all sequence pairs within each group were analyzed. Analysis was conducted using the Tamura 3-parameter model. The rate variation among sites were modeled with a gamma distribution (shape parameter = 1). The analysis involved 15 nucleotide sequences. Analyses were conducted in MEGA6. The variable substitution site was calculated by DAMBE: Software Package [13].

### Statistical analysis

Hardy-Weinberg equilibrium by a chi-square (χ2) test with one degree of freedom (df) was performed between case and control subjects. Fisher’s exact test was also used for comparing the demographic and habits between patients and controls. The polymorphism and demographic factor in each group were estimated for their association with diabetes using odds ratios (ORs) and 95 % confidence intervals (CIs) in the Logistic Regression (LR) Model adjusted with multivariable analysis. Each polymorphism was checked by the presence and absence of the SNPs. Additionally, logistic regression analyses were conducted to compute the influence of both genetic and environmental factors. For all tests, a two-sided *P*-value <0.05 was considered statistically significant. All statistical analyses were performed using SPSS 20.0 program (SPSS Ibérica, Madrid, Spain) and SYSTAT 13.0. (Systat Software Inc., USA).

## Results

In the present study, blood samples of 58 Diabetic patients and 50 healthy individuals were analyzed. The prevalence of Type 2 diabetes was higher in patients who consumed smoked meat and excess fat (Odd Ratio, OR: 4.76, 95 % Confidence Interval, 95 % CI: 1.03–13.73). This was observed especially among men (OR: 1.14, 95 % CI: 0.25–1.63). Smoking, consuming betel-nut with paan and alcohol were major risk factors for type 2 diabetes. In Mizo population, Type 2 diabetes is not significantly associated with familial history. There are significant differences in the age of onset (*p* < 0.001) of diabetes and there was no significant differences in gender or concentration of plasma glucose level (Additional file 1: Table S1). As is typical for Hardy-Weinberg equilibrium, the degree of significance was quite coarse due to the less number of sample size using both chi-square (χ2) and fisher’s exact tests.

Total of 6 ATPase (ATP6 and ATP 8) and ND1 sequence variations at six distinct nucleotide positions were found in 21.8 and 54.54 % samples, respectively. One heteroplasmic variation (MT3970 C > T) was found in 36.36 % of samples (EBI Accession No. LN558438 - LN558467and NDI regions - LN558501 - LN558513). In the ATPase and ND1 genes, 6 non-synonymous and 6 synonymous substitutions were found and out of these, 3 non-synonymous and 2 synonymous were not previously reported in the literature or the public mtDNA mutation databases (mtDBase:http://www.genpat.uu.se/mtDB/index.html; MITOMAP:http://mitomap.org/ MITOMAP) related with diabetes (Table 1).

**Table 1:** Polymorphisms in ATPase and ND1 genes of diabetic samples in Mizo population

Gene name	Frequency of mutation^a^ (%)	Reference nucleotide	Nucleotide change	Nomenclature of mutation	Codon number	Codon position	Syn/Non syn	Codon change	Amino acid substitution	Reported/Disease caused	Polyphen2 score
ATP8	9.09	C	C > T	8414 C > T	17	1	Non-Syn	CTC > TTC	L > F	Reported for prostate cancer. Not for diabetes	0.99 (probably damaging)
ATP6	18.18	G	G > A	8584 G > A	20	1	Non-Syn	GCA > ACA	A > T		0.073 (Benign)
	9.09	T	T > C	8602 T > C	26	1	Non-syn	TTT > CTT	F > L		0.015 (Benign)
	9.09	G	G > A	8616 G > A	30	3	Syn	TTG > TTA	L > L	Reported for normal variation	---
	18.18	A	A > G	8701 A > G	59	1	Non-syn	ACC > GCC	T > A	Reported for diabetes	0.002 (Benign)
	9.09	G	G > A	8790 G > A	88	3	Syn	CTG > CTA	L > L	Reported for breast cancer. Not for Diabetes	---
ND1	9.09	G	G > A	3316 G > A	4	1	Non-syn	GCC > ACC	A > T	Reported for diabetes	0.00 (Benign)
	9.09	T	T > C	3394 T > C	30	1	Non-syn	TAT > CAT	Y > H		0.021 (Benign)
	9.09	T	T > A	3552 T > A	82	3	Syn	GCT > GCA	A > A		---
	36.36	C	C/T > T/C	3970 C > T	222	1	Syn	CTA > TTA	L > L		---
	9.09	A	A > G	4065 A > G	253	3	Syn	GAA > GAG	E > E		---
	27.27	C	C/T > T/C	4149 C > T	281	3	Syn	CGC > CGT	R > R	Reported for breast cancer	---

^a^Mutation frequency was calculated based on the total number of mutations obtained against the total number of cases

Genetic divergence was estimated between the diabetic and healthy samples by calculating the number of base substitutions. Divergence distance between the diabetic and healthy samples were found to be 0.002 for ATP6, nil for ATP 8 and 0.002 for ND1 genes. The diabetic and healthy samples clustered in two separate clades for ATP6 and ND1 gene sequences (data not shown). Majority of the nucleotide diversity were present in the start region for ATP6 and both start and end regions for NDI gene. The transition for all the three genes is higher than transversion. The transition- transversion bias for the ATP6 is high for diabetes and healthy control samples (Table 2). The haplotype frequency P, Watterson’s Θ, which compares two estimators of the population parameter is higher in ATP8 gene than NDI and ATP8. Based on the Tajima test statistics, ATP6 gene is a good marker for genetic variation analysis between the healthy and diabetic samples (Table 2).

**Table 2:** Characteristic features of healthy and diabetic samples

Gene name	Nucleotide frequency (GC) %		Transition/Transversion bias (R)		∏		Θ		S		Ps		D	
	H	D	H	D	H	D	H	D	H	D	H	D	H	D
ATP6	44.3	44.4	132.25	151.55	0.0015	0.0021	0.0016	0.0025	11	2	0.0016	0.0073	−0.7099	−0.6484
ATP8	39.6	39.6	0.39	293.17	0.0000	0.0009	0.0000	0.0016	0	1	0.0000	0.0048	n/c	1.1285
ND1	47.7	47.7	0	2.23	0.0007	0.0017	0.0006	0.0015	1	4	0.0010	0.0042	1.6329	−0.3849

*Abbreviations*: *S* number of segregating sites, *ps* S/n, *Θ* ps/a1, *π* nucleotide diversity, *D* Tajima test statistic, *H* healthy, *D* diabetic

## Discussion

The main objective of the study was to find out the role of demographic factors in the onset of diabetes and to detect their associated mutations in mitochondrial genes in a lesser known Mizo population. The prevalence of T2D rise with family history of diabetes and clinical representation depends mostly on the severity of insulinopenia, lack of physical activity, obesity, demographic factor and involvement of genetic factors [14]. From this study, the risk for T2DM was found to be higher in patients with high smoked meat consumption followed by excess smoking and alcoholism. Saum, which is fermented pork fat, is one of the favorite foods of the Mizo people and is rich in hydrogenated oils. We also assessed the correlation between mtDNA gene mutations withrecognized prognostic relevance. Mitochondria play an imperative role in glucose metabolism, insulin secretion and biogenesis, hence its dysfunction is reportedly found to play a crucial role in diabetes development [2, 15]. Earlier reports showed the association of mitochondrial DNA mutations like 1310C < T, 1382A < C, 1438G < A, 1201A < G, 3243A < G, 3252A < G, 3256A < T, 3264A < C, 3271A < C, 3290T < C, 3303C < T, 3316G < A, 3394T < C, 8296A < G, 8344A < G, 11778G < A, 12026A < G, 12258C < A, 14577T < C, 14709T < C and 16189T < C [16–20] with T2D development. Particularly, mutation in tRNA Leu gene at 3243 (A < G) position and in the subunits of NADH dehydrogenase 1 and 4 have been reported to have strong association with incidence of diabetes in different populations [18, 19, 21, 22]. There was a significant correlation between the number of somatic mtDNA ATP6 mutations and the smoking and consuming betel-nut with paan (OR: 3.52; 95 % CI: 0.96–12.11) for the type 2 diabetes along with drinking alcohol (OR: 4.62; 95 % CI: 1.82–14.53). Besides, there was no significant difference in the concentration of plasma glucose level and familial history among the diabetic patients. Epidemic evidence have suggested that chronic smokers or tobacco consumers have a higher risk to be insulin resistant and exhibiting several aspects of the insulin resistance syndrome leading to the development of T2DM [23]. Earlier studies have identified a point mutation in the mitochondrial gene in a family with slowly progressive insulin-dependent diabetes mellitus (IDDM) or insulin-deficient non-IDDM. They have identified A to G transition at 3243 occurring in a highly conserved region of the tRNALeu (UUR) gene and this SNP and diabetes mellitus are maternally inherited and co-segregated [24–26].

High risk diabetic factors were seen in people with old age group belonging to low economic status with excessive meat intake and mostly prevalent among men. Obesity may also play an important role in triggering T2DM. History of familial inheritance is rarely seen in the case of Mizo Population. The Prevalence of Diabetes in India Study (PODIS) was carried out in 108 centres (49 urban and 59 rural) in different parts of India to look at the urban–rural differences in type 2 diabetes and glucose intolerance in the year 2004 [27, 28]. Our report is the first mitochondrial genetic alterations in diabetes reported from Mizo-Mongloid population.

Our results also revealed that non-synonymous variations were more frequent in the ATPase than in ND1 region of diabetic patients. ATP 6 belonging to ATP gene family is more mutated in this case than ATP 8. This indicates that simple sampling of blood would be advantageous for early marker development. The studied genes undergoes transitional substitution rather than transversion. Moreover, Tajima’s D statistical testshows that ATP6mtDNA gene evolves randomly and NDI gene is evolving under a non-random process. This might depend on the directional selection or balancing selection or demographic expansion for Mizo population.

In our study, one major limitation is the small sample size which resulted in unstable risk estimates with wide 95 % CIs. The rate and standard deviation of mutation frequencies decreased with increasing sample size. There is a point beyond which increased sampling will have little impact on the accuracy and precision of estimates of mutation frequency. The risk estimates for ORs of diabetes in relation to lifestyle factors might have been biased, due to a small sample size and other factors such as selection bias. Another limitation of our study is that it does not explain the mitochondrial maternal inheritance of the mutations, because this study does not contain any familial sample.

## Conclusion

To our knowledge, the present study is a novel finding in terms of the possible role of mtDNA ATPase and ND1 mutations in T2DM. ATP6 can be a good marker for the early detection of type 2 diabetes in Mizo-Mongoloid population. The mitochondrial gene alterations may attribute for diabetes risk along with the demographic habits and diet in Mizoram, Northeast Indian population. Besides clinical inconsistency, socio-economic status and environmental information needs to be considered in the assessment of risk profile of diabetic patients by health service.

### Ethics, consent and permissions

The ethical committee of all institutes (Civil Hospital and Mizoram University, Aizawl, Mizoram, India) approved the study protocol involved in the study. All volunteers were fully informed about the study and participated with their full consent.

## Additional file

Additional file 1: Table S1. Demographic and biochemical profiles of the diabetic patient samples from Mizo population. (DOC 106 kb)
