# Supplementary material for: Mitochondrial complex I and V gene polymorphisms in type II diabetes mellitus among high risk Mizo-Mongoloid population, Northeast India
Source: Genes Environ. 2016 Mar 1;38:5. doi: 10.1186/s41021-016-0034-z (PMC4917945; doi:10.1186/s41021-016-0034-z)
Supplement: Additional file 1: Table S1. — Demographic and biochemical profiles of the diabetic patient samples from Mizo population. (DOC 106 kb) [file 41021_2016_34_MOESM1_ESM.doc]

**Table S1.** Demographic and Biochemical profiles of the diabetic patient samples from Mizo population

| **Sex** | **Age**  **(yrs)** | **Diet Information** | | | | | | **Tobacco Product information** | | | | **Alcohol Intake** | **Familial**  **Information** | **Biochemical Profiles (mg/dl)** | | | | | |
| --- | --- | --- | --- | --- | --- | --- | --- | --- | --- | --- | --- | --- | --- | --- | --- | --- | --- | --- | --- |
|  |  | **Meat** | **Fat** | | **Saum** | **Smoked Meat** | | **Smoking** | **KUHVA** | | **SAHDAH** |  |  | **Fasting** | **Post Prandial** | | **Cholesterol/lipid** | **Creatinine** | |
| F | 48 | +++ | | + | ++ | | ++ | - | | + | + | - | - | 99 | | 131 | 152 | | 0.7 |
| M | 70 | +++ | | + | +++ | | +++ | - | | - | + | - | - | 107 | | 229 | 182 | | 1.1 |
| M | 38 | +++ | | + | + | | +++ | + | | - | - | + | + | 90 | | 114 | 176 | | 1.5 |
| M | 66 | +++ | | ++ | ++ | | ++ | - | | - | + | - | - | 278 | | 471 | 129 | | 0.9 |
| F | 50 | +++ | | + | + | | +++ | - | | - | + | - | - | 99 | | 103 | 115 | | 1.6 |
| M | 79 | +++ | | + | ++ | | ++ | - | | + | + | - | - | 122 | | 172 | 109 | | 1.3 |
| F | 48 | +++ | | + | ++ | | ++ | - | | + | ++ | - | + | 115 | | 189 | 167 | | 0.8 |
| M | 67 | ++ | | +++ | ++ | | +++ | + | | + | + | + | - | 128 | | 358 | 164 | | 1 |
| M | 73 | +++ | | ++ | + | | +++ | + | | + | + | + | - | 108 | | 269 | 182 | | 1.5 |
| F | 49 | ++ | | +++ | + | | ++ | - | | + | ++ | - | + | 330 | | 450 | 146 | | 1.2 |
| F | 72 | ++ | | + | ++ | | ++ | - | | - | + | - | - | 122 | | 239 | 175 | | 0.8 |
| M | 52 | ++ | | ++ | + | | ++ | - | | - | + | + | - | 135 | | 168 | 135 | | 1 |
| M | 52 | ++ | | ++ | ++ | | ++ | - | | - | ++ | + | - | 125 | | 265 | 146 | | 1.4 |
| F | 38 | ++ | | + | + | | + | + | | + | ++ | - | + | 222 | | 260 | 189 | | 1 |
| F | 40 | +++ | | + | ++ | | +++ | + | | + | ++ | - | + | 90 | | 177 | 198 | | 1 |
| F | 64 | +++ | | ++ | ++ | | + | - | | + | + | - | - | 120 | | 272 | 173 | | 0.6 |
| F | 58 | ++ | | + | + | | +++ | - | | + | + | - | - | 143 | | 228 | 146 | | 0.8 |
| M | 81 | + | | + | + | | +++ | - | | - | - | - | - | 116 | | 171 | 157 | | 0.7 |
| F | 50 | +++ | | + | ++ | | +++ | - | | + | ++ | - | - | 107 | | 165 | 138 | | 1.4 |
| M | 52 | +++ | | + | + | | +++ | - | | + | + | + | + | 233 | | 356 | 202 | | 0.9 |
| M | 68 | +++ | | - | + | | ++ | - | | + | + | + | - | 116 | | 250 | 163 | | 1 |
| F | 37 | ++ | | - | ++ | | ++ | + | | + | ++ | - | + | 142 | | 270 | 166 | | 0.8 |
| M | 38 | +++ | | - | + | | ++ | + | | + | - | + | + | 125 | | 316 | 184 | | 1 |
| M | 59 | ++ | | + | + | | ++ | + | | + | + | + | + | 139 | | 270 | 159 | | 1.3 |
| M | 80 | + | | ++ | + | | ++ | + | | - | + |  | - | 199 | | 235 | 172 | | 1.5 |
| F | 64 | +++ | | ++ | + | | ++ | - | | - | + | - | - | 108 | | 120 | 164 | | 1.8 |
| F | 58 | ++ | | + | + | | ++ | - | | + | + | - | - | 90 | | 105 | 177 | | 0.6 |
| M | 57 | +++ | | + | + | | ++ | + | | + | + | + | - | 90 | | 201 | 225 | | 0.5 |
| M | 53 | +++ | | + | + | | ++ | + | | + | + | + | - | 238 | | 325 | 128 | | 1.6 |
| F | 40 | +++ | | + | + | | ++ | + | | + | ++ | - | + | 137 | | 151 | 168 | | 1 |
| F | 43 | ++ | | + | + | | ++ | - | | + | ++ | - | + | 80 | | 95 | 137 | | 0.8 |
| M | 49 | +++ | | + | + | | ++ | + | | + | + | + | + | 137 | | 131 | 142 | | 0.6 |
| F | 62 | +++ | | + | + | | ++ | - | | - | + | - | - | 127 | | 199 | 185 | | 1.2 |
| F | 55 | ++ | | ++ | ++ | | ++ | - | | - | ++ | - | - | 95 | | 295 | 167 | | 1.4 |
| M | 48 | +++ | | + | + | | +++ | + | | + | + | ++ | - | 252 | | 336 | 134 | | 1.8 |
| M | 60 | ++ | | + | + | | +++ | + | | - | + | + | - | 194 | | 425 | 194 | | 1 |
| M | 62 | ++ | | + | + | | ++ | + | | - | + | + | - | 104 | | 201 | 127 | | 0.6 |
| M | 60 | ++ | | + | + | | +++ | + | | - | + | + | - | 117 | | 140 | 168 | | 0.8 |
| M | 65 | ++ | | + | + | | ++ | + | | - | + | - | - | 296 | | 470 | 129 | | 1 |
| F | 45 | +++ | | + | + | | ++ | - | | + | ++ | - | + | 95 | | 216 | 235 | | 1 |
| F | 85 | + | | + | + | | ++ | - | | - | + | - | - | 112 | | 277 | 211 | | 1 |
| M | 42 | +++ | | + | + | | ++ | + | | - | + | + | + | 174 | | 189 | 148 | | 1 |
| M | 54 | +++ | | ++ | + | | +++ | + | | - | - | + | - | 68 | | 170 | 155 | | 1 |
| F | 74 | ++ | | + | + | | +++ | - | | - | + | - | - | 79 | | 231 | 174 | | 0.9 |
| F | 57 | +++ | | + | + | | +++ | - | | - | + | - | - | 158 | | 251 | 194 | | 0.7 |
| M | 62 | +++ | | + | + | | +++ | + | | - | + | + | - | 80 | | 175 | 191 | | 0.8 |
| M | 61 | ++ | | + | + | | +++ | + | | - | + | + | - | 141 | | 303 | 173 | | 0.6 |
| F | 46 | ++ | | - | + | | +++ | + | | + | + | - | + | 109 | | 145 | 166 | | 0.6 |
| F | 58 | ++ | |  | + | | +++ | - | | - | ++ | - | - | 89 | | 212 | 133 | | 1.2 |
| M | 48 | +++ | | - | + | | ++ | + | | - | + | + | + | 199 | | 356 | 105 | | 1 |
| M | 66 | +++ | | + | + | | ++ | + | | - | + | - | - | 80 | | 190 | 241 | | 0.8 |
| F | 43 | ++ | | + | + | | +++ | - | | + | + | - | + | 90 | | 256 | 153 | | 0.7 |
| F | 35 | ++ | | + | + | | +++ | - | | + | ++ | - | + | 177 | | 287 | 142 | | 1.4 |
| M | 60 | +++ | | + | + | | +++ | + | | + | + | - | - | 108 | | 215 | 168 | | 0.5 |
| F | 64 | +++ | | + | + | | +++ | - | | - | + | - | - | 119 | | 133 | 118 | | 1.5 |
| F | 57 | ++ | | + | + | | +++ | - | | - | + | - | - | 107 | | 112 | 225 | | 1.6 |
| F | 39 | ++ | | + | + | | +++ | - | | + | ++ | - | + | 101 | | 111 | 102 | | 1 |
| F | 22 | ++ | | + | + | | ++ | - | | + | - | - | + | 80 | | 100 | 111 | | 1 |

+ low; ++ medium; +++ High consumption; Familial Information: + Present; - Absent.
